# Arm Selection Preference of MicroRNA-193a Varies in Breast Cancer

**DOI:** 10.1038/srep28176

**Published:** 2016-06-16

**Authors:** Kuo-Wang Tsai, Chung-Man Leung, Yi-Hao Lo, Ting-Wen Chen, Wen-Ching Chan, Shou-Yu Yu, Ya-Ting Tu, Hing-Chung Lam, Sung-Chou Li, Luo-Ping Ger, Wen-Shan Liu, Hong-Tai Chang

**Affiliations:** 1 Department of Medical Education and Research, Kaohsiung Veterans General Hospital, Kaohsiung, Taiwan; 2 Department of Chemical Biology, National Pingtung University of Education, Pingtung, Taiwan; 3 Department of Radiation Oncology, Kaohsiung Veterans General Hospital, Kaohsiung, Taiwan; 4 Department of Family Medicine, Zuoying Branch of Kaohsiung Armed Forces General Hospital, Kaohsiung, Taiwan; 5 Molecular Medicine Research Center, Chang Gung University, Taoyuan, Taiwan; 6 Bioinformatics Center, Chang Gung University, Taoyuan, Taiwan; 7 Genomics & Proteomics Core Laboratory, Department of medical research, Kaohsiung, Chang Gung Memorial Hospital and Chang Gung University College of Medicine, Kaohsiung, Taiwan; 8 Center For Geriatrics and Gerontology, Kaohsiung Veterans General Hospital, Kaohsiung, Taiwan; 9 Institute of Biomedical Sciences, National Sun Yat-Sen University, Kaohsiung, Taiwan; 10 Department of Radiation Oncology, Tri-Service General Hospital, Taipei, Twiwan; 11 Department of Surgery, Kaohsiung Veterans General Hospital, Kaohsiung, Taiwan

## Abstract

MicroRNAs (miRNAs) are short noncoding RNAs derived from the 3′ and 5′ ends of the same precursor. However, the biological function and mechanism of miRNA arm expression preference remain unclear in breast cancer. We found significant decreases in the expression levels of miR-193a-5p but no significant differences in those of miR-193a-3p in breast cancer. MiR-193a-3p suppressed breast cancer cell growth and migration and invasion abilities, whereas miR-193a-5p suppressed cell growth but did not influence cell motility. Furthermore, NLN and CCND1, PLAU, and SEPN1 were directly targeted by miR-193a-5p and miR-193a-3p, respectively, in breast cancer cells. The endogenous levels of miR-193a-5p and miR-193a-3p were significantly increased by transfecting breast cancer cells with the 3′UTR of their direct targets. Comprehensive analysis of The Cancer Genome Atlas database revealed significant differences in the arm expression preferences of several miRNAs between breast cancer and adjacent normal tissues. Our results collectively indicate that the arm expression preference phenomenon may be attributable to the target gene amount during breast cancer progression. The miRNA arm expression preference may be a means of modulating miRNA function, further complicating the mRNA regulatory network. Our findings provide a new insight into miRNA regulation and an application for breast cancer therapy.

Breast cancer is one of the major causes of cancer-related deaths worldwide and the most common cancer among women^1^. Metastasis to distant organs and lymph nodes is a major problem that usually results in high mortality. Investigating breast cancer-associated genes for early detection or therapeutic targeting can improve the survival rates of breast cancer patients.

MicroRNAs (miRNAs) are small RNA molecules with critical regulatory functions in several physiological processes^2^. Mature miRNAs are produced from primary transcripts (pri-miRNAs) via 2 maturation steps. Precursor miRNAs (pre-miRNAs), which are approximately 70 nucleotides in length and composed of a 5p arm, 3p arm, and terminal loop, are generated from Drosh-processed pri-miRNAs. Subsequently, these stem-loop structures (pre-miRNAs) are exported to the cytoplasm by exportin 5, where they are cleaved by Dicer to release the terminal loop and the 5p–3p duplex. Finally, the miR#-5p or miR#-3p arm is selectively loaded onto the RNA-induced silencing complex (RISC) and targets the 3′UTR of the target genes^3^,^4^,^5^. The selection of mature miRNA (5p or 3p arm) is determined by the Ago protein on the basis of the hydrogen bonding selection mechanism; this protein cleaves the complementary miR#-5p–miR#-3p duplex to facilitate loading of a mature miRNA strand onto the RISC [17–18]. Recent studies have reported that miR#-5p and miR#-3p arms can be preferentially selected among different tissues, developmental stages, and species and during cancer progression^6^,^7^,^8^,^9^,^10^,^11^,^12^,^13^,^14^. Our previous studies have indicated that the arm selection of some miRNAs significantly varies among human cancers, including hepatocellular carcinoma, gastric cancer, and breast cancer^8^,^9^,^14^. Therefore, the simple thermodynamic hydrogen bonding theory is insufficient to explain the phenomenon of flexible selection known as arm switching or arm selection preference.

Previous studies have revealed that miR-193a expression is silenced by DNA hypermethylation in human cancers^15^,^16^,^17^,^18^,^19^. According to miRbase data, 2 mature miRNAs can be generated from pri-miR-193a: a dominant arm (miR-193a-3p) and a passenger arm (miR-193a-5p). Numerous studies have reported that miR-193a-3p suppresses tumour development by silencing SRSF2, HIC2, HOXC9, PSEN1, LOXL4, ING5, c-kit and PLAU, and MCL-1 in human cancers^15^,^16^,^20^,^21^,^22^,^23^,^24^,^25^,^26^,^27^,^28^.

A few studies have also revealed that miR-193a-5p suppresses tumour development by modulating cancer cell growth^27^,^29^. A previous study reported that miRNA may play contrasting dual functions depending on its target gene expression in different cancer types^30^. However, the expression mechanism and biological function of miR-193a-5p and miR-193a-3p remain unclear in breast cancer. In this study, we assessed the roles of miR-193a-5p and miR-193a-3p in breast cancer by using bioinformatics and experimental approaches.

## Results

### Different arm expression preferences of miR-193a in breast cancer

**Figure 1:**
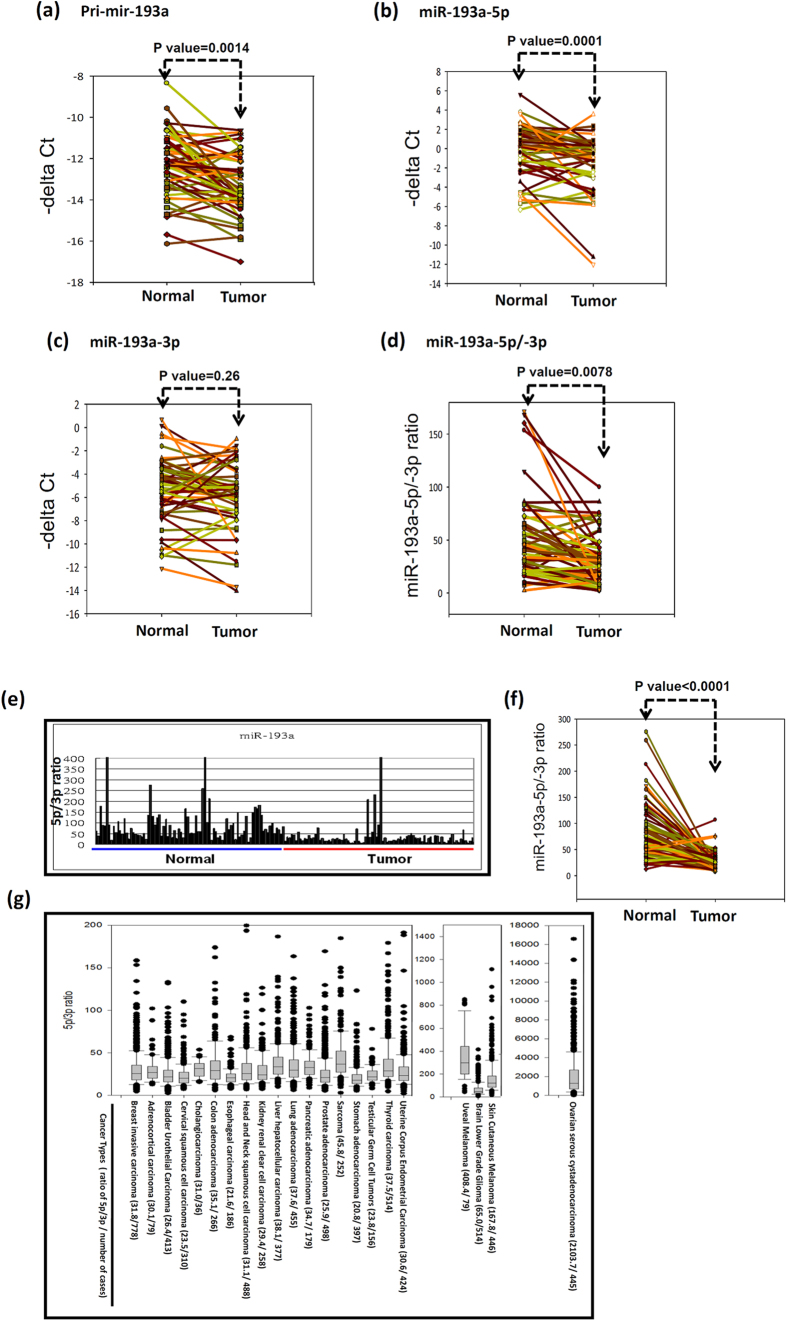
Expression of miR-193a-5p and miR-193a-3p significantly changed during breast cancer carcinogenesis or varied among cancer types. (**a**) The expression levels of pri-mir-193a were examined in breast cancer by using real-time PCR. (**b**,**c**) The expression levels of miR-193a-5p and miR-193a-3p were compared between breast cancer and adjacent normal tissues from 53 patients. (**d**) The miR-193a-5p/miR-193a-3p ratio was compared between 53 breast cancer tissues and corresponding adjacent normal tissues. (**e**,**f**) The expression levels of miR-193a-5p and miR-193a-3p were obtained from TCGA database. Significant differences were observed in the miR-193a-5p/miR-193a-3p ratios between breast cancer and adjacent normal tissues from 87 patients. The data of pri-mir-193a, miR-193a-5p or miR-193a-3p expression in breast tissues were analyzed using paired t test. (**g**) The miR-193a-5p/miR-193a-3p ratios were assessed in various human cancers. Data were analysed using the Student *t* test (*P* < 0.05 was considered significant).

The expression levels of primary miR-193a were significantly decreased in breast cancer tissues compared with those in corresponding adjacent normal tissues (Fig. 1a). Furthermore, we examined the expression levels of mature miR-193a and observed that only miR-193a-5p (*p* value < 0.001) was significantly downregulated in breast cancer tissues (Fig. 1b). No differences were observed in the expression levels of miR-193a-3p between breast cancer and adjacent normal tissues (Fig. 1c). As shown in Fig. 1d, the miR-193a-5p/miR-193a-3p ratio was significantly decreased in breast cancer tissues compared with that in adjacent normal tissues (*p* value = 0.0078). This result revealed changes in the arm selection preference of miR-193a during the miRNA maturation process. Using TCGA data, we also observed a significant difference in the expression preference of miR-193a-5p and miR-193a-3p between breast cancer and corresponding adjacent normal tissues (Fig. 1e,f). Analysis of TCGA database revealed significant differences in the arm expression preference of miR-193a among cancer types. Particularly, the miR-193-5p/miR-193-3p ratio was apparently high in ovarian cancer, melanoma, and glioma (Fig. 1g). These data indicated changes in the expression preference of miR-193a-5p and miR-193a-3p during the miRNA maturation process in breast cancer.

### miR-193a-5p and miR-193a-3p contribute to breast cancer cell growth and motility

**Figure 2:**
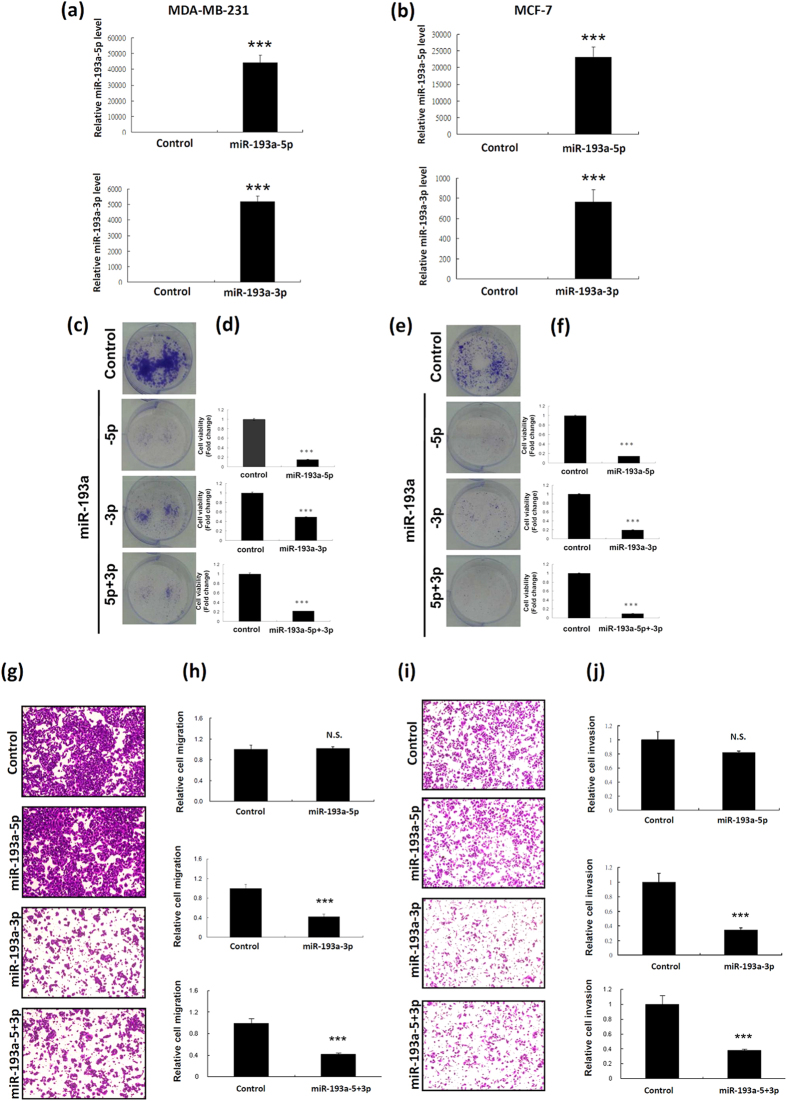
Ectopic expression of miR-193a-5p and miR-193a-3p affected breast cancer cell growth, migration, and invasion. (**a**,**b**) Relative levels of miR-193a-5p and miR-193a-3p in MDA-MB-231 and MCF-7 cells were analysed through stem-loop RT-qPCR after transfection with miR-193a-5p or miR-193a-3p mimics. (**c**–**f**) The colony formation assay was performed after transfection of MDA-MB-231 or MCF7 cells with miR-193a-5p or miR-193a-3p mimics, or both miR-193a-5p and miR-193a-3p mimics for 2 wk. The cell images of a representative experiment are shown (**c**,**e**), and a graph shows quantified values (**d**,**f**). Data are reported as the number of colonies relative to the control (means ± SD). ***P < 0.001 vs control. (**g**–**j**) The migration and invasion abilities of MDA-MB-231 cells transfected with miR-193a mimics for 24 h were assessed using the Transwell assay. Subsequently, cells were stained with crystal violet solution, and the numbers of cells that migrated were quantified by counting the cells in 3 fields under a phase-contrast microscope. The cell images of a representative experiment are shown, and the graph shows values quantified using Ascent software. Data are reported as the number of colonies relative to the control (means ± SD). ***P < 0.001 vs control.

To understand the individual biological functions of miR-193a-5p and miR-193a-3p in breast cancer, the miR-193a-5p and miR-193a-3p mimics were separately transfected into breast cancer cells, and then the cell growth, migration, and invasion were examined. As shown in Fig. 2a,b, the expression levels of miR-193a-5p and miR-193a-3p significantly increased after transfection with the mimics. The ectopic expression of miR-193a-5p and miR-193a-3p significantly suppressed breast cancer cell colony formation (Fig. 2c–f). MiR-193a-3p overexpression suppressed MDA-MB-231 migration and invasion abilities, whereas miR-193a-5p did not influence breast cancer cell migration (Fig. 2g,h) and invasion ability (Fig. 2i,j). These results indicated that miR-193a-5p and miR-193a-3p exhibited different biological functions in breast cancer cells.

### Examination of target genes of and pathways regulated by miR-193a-5p and miR-193a-3p

**Figure 3:**
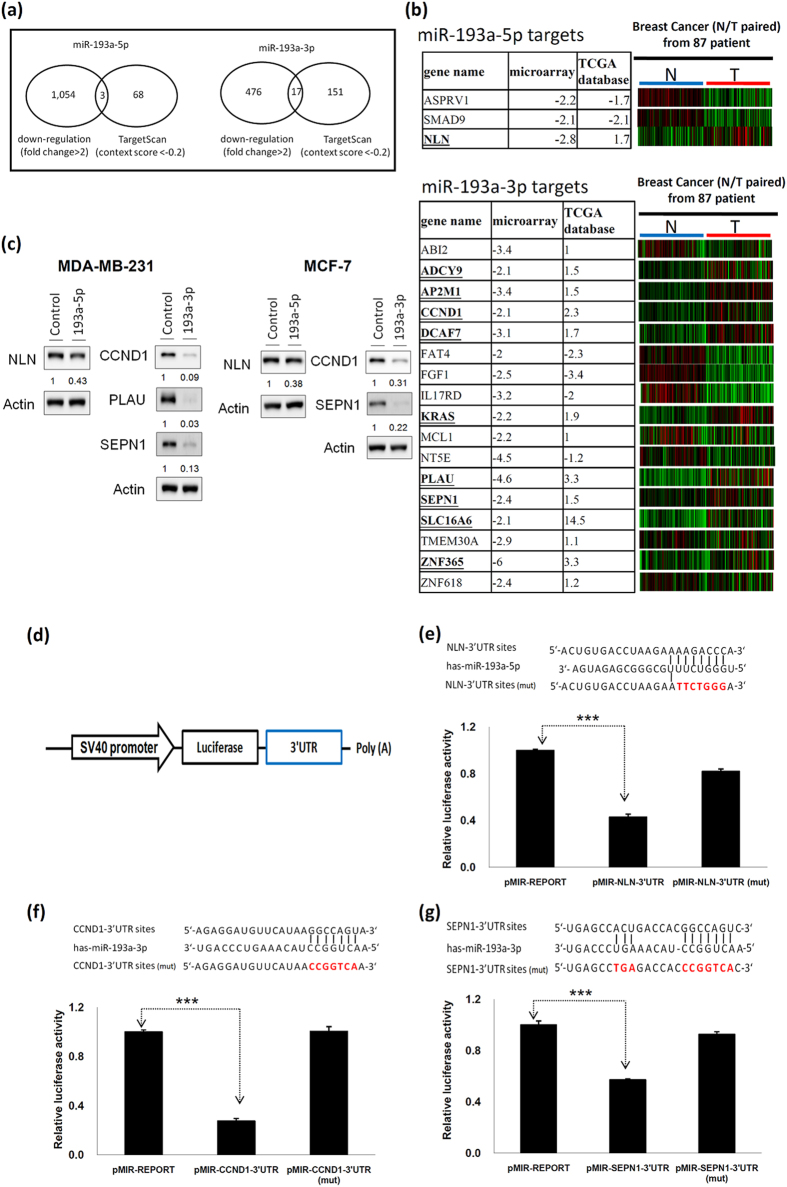
Identification of direct target genes of miR-193a-5p and miR-193a-3p in breast cancer cells. (**a**) Venn diagrams showing the numbers of target genes of miR-193a-5p and miR-193a-3p that were identified using the TargenScan tool and the microarray approach. (**b**) The expression levels of potential targets were analysed in breast cancer tissues in comparison with those in corresponding normal tissues from 87 breast cancer patients; these tissues were obtained from TCGA data set. (**c**) The proteins encoded by target genes of miR-193a-5p and miR-193a-3p were examined after transfection of MDA-MB-231 and MCF-7 cells with miR-193a-5p and miR-193a-3p mimics. (**d**) Schema of the luciferase constructs. The miR-193a target sequence in the 3′UTR region of target genes is shown in the upper panel and the mutant of its 3′-UTR is shown in red. Relative luciferase activity of the reporter with the 3′UTR and 3′UTR(mut) of NLN (**e**), CCND1 (**f**), and SEPN1 (**g**) genes was determined after cotransfection of breast cancer cells with miR-193a-5p or miR-193a-3p mimics. Firefly luciferase activity served as a normalization control.

To clarify the mechanism involved in suppression of MDA-MB-231 cell growth and invasion ability by miR-193a-5p and miR-193a-3p, we identified their direct target genes by using microarray and bioinformatics approaches. We used the microarray approach to determine the transcriptome profile of miR-193a-5p and miR-193a-3p mimic-transfected cells. According to the transcriptome data, 1,054 and 476 genes were downregulated by more than 2-fold after miR-193a-5p and miR-193a-3p transfection for 48 h, respectively. In addition, using the TargetScan prediction tool, we identified that the 3′UTR region of 68 and 151 putative target genes may be targeted by miR-193a-5p and miR-193a-3p, respectively (Fig. 3a). Combining data from microarray experiments and the target prediction tool, we identified 3 putative target genes for miR-193a-5p and 17 target genes for miR-193a-3p. Furthermore, both miR-193a-5p and miR-193a-3p suppress tumour development by inhibiting breast cancer cell growth. Therefore, the target genes of miR-193a should have oncogenic functions and be overexpressed in breast cancer. The expression levels of these putative target genes in breast cancer tissue from 87 patients were obtained from TCGA database. According to the aforementioned criteria, NLN is a putative target gene for miR-193a-5p, and several putative direct target genes (ADCY9, AP2M1, CCND1, DCAF7, KRAS, PLAU, SEPN1, SLC16A6, and ZNF365) can be targeted by miR-193a-3p. Real-time PCR for confirming these target genes showed that the expression levels of NLN were significantly suppressed in miR-193a-5p mimic-transfected cells, and those of CCND1, DCAF7, PLAU, and SEPN1 were downregulated in miR-193a-3p mimic-transfected MB-231 and MCF7 cells (Supplementary Fig. S1a,S1b). MiR-193a-3p mimics significantly silenced the expression of the CCND1, PLAU, and SEPN1 proteins, but not the DCAF7 protein, in transfected cells (Fig. 3c and Supplementary Fig. S1c). MiR-193a-5p significantly silenced the expression of the NLN protein in breast cancer cells (Fig. 3c). We assayed luciferase activity to assess whether these candidates are direct targets of miR-193a-5p or miR-193a-3p. Our data indicated that miR-193a-5p suppressed the luciferase activity of pMIR-NLN-3′UTR, whereas miR-193a-3p silenced the luciferase activity of pMIR-CCND1-3′UTR, pMIR-PLAU-3′UTR, and pMIR-SEPN1-3′UTR (Fig. 3d–g and Supplementary Fig. S2). Furthermore, the luciferase activity of pMIR-NLN-3′UTR(mut), pMIR-CCND1-3′UTR(mut), pMIR-PLAU-3′UTR(mut), and pMIR-SEPN1-3′UTR(mut) was not brought down in the mimic transcfection cells (Fig. 3f–g, and Supplementary Fig. S2). In summary, NLN expression was directly regulated by miR-193a-5p, and CCND1, PLAU, and SEPN1 expression was directly regulated by miR-193a-3p in breast cancer cells.

### Target genes of miR-193a involved in cell growth, migration, and invasion

**Figure 4:**
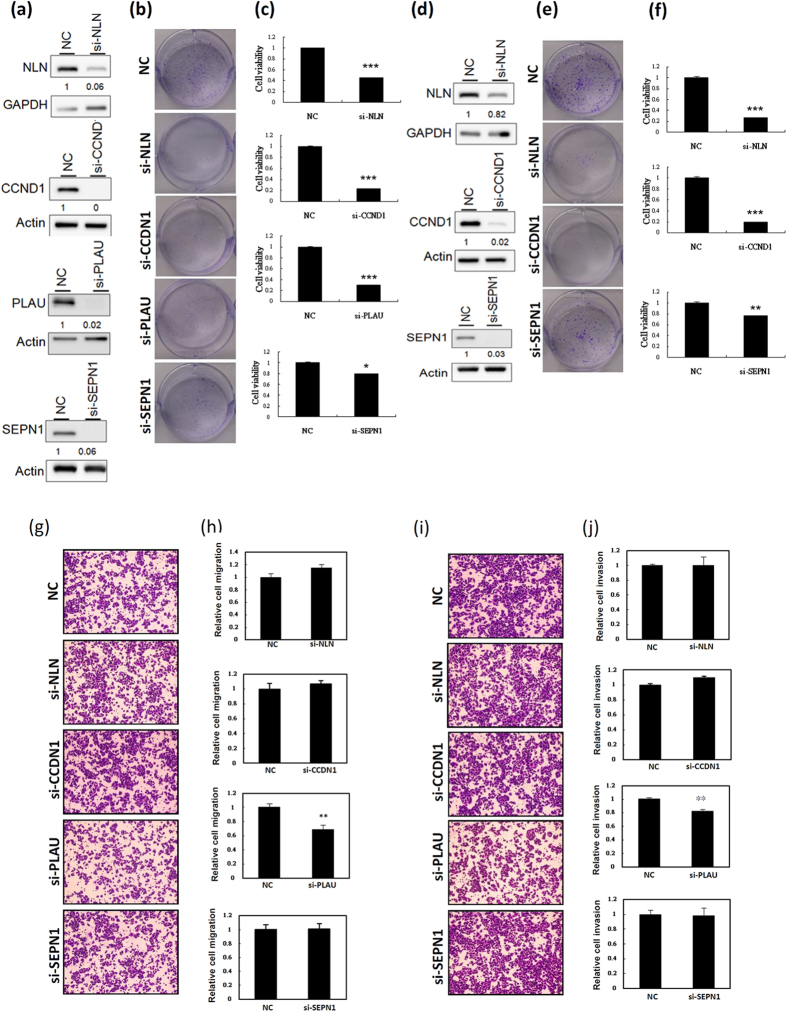
Knockdown of the expression of direct targets of miR-193a-5p and miR-193a-3p suppressed growth and motility of breast cancer cells. (**a**) The expression levels of target genes were examined after transfection of MDA-MB-231 cells with siRNA. (**b**) The colony formation assay was performed after transfection of MDA-MB-231 cells with si-NLN, si-CCND1, si-PLAU, and si-SEPN1 for 2 wk, and a graph shows quantified values (**c**,**d**) The expression levels of target genes in MCF-7 cells were examined after siRNA transfection. (**e**) The colony formation assay was performed after transfection of MCF-7 cells with si-NLN, si-CCND1, and si-SEPN1 for 2 wk, and a graph shows quantified values (**f**–**j**) The migration and invasion abilities were assessed using the Transwell assay after transfection of MDA-MB-231 cells with siRNA. The cell images of a representative experiment are shown, and the graph shows values quantified using Ascent software. Data was reported as the number of colonies relative to the control (means ± SD). ***P < 0.001 vs control.

We examined the function of the target genes of miR-193a-5p and miR-193a-3p involved in breast cancer cell growth and motility. As shown in Fig. 4a–f, knockdown of CCND1, PLAU, SEPN1, and NLN suppressed cell growth, similar to miR-193a-5p and miR-193a-3p overexpression in breast cancer cells. However, only PLAU knockdown significantly suppressed the migration and invasion abilities of MDA-MB-231 cells (Fig. 4g–j). Knockdown of NLN, CCND1, and SEPN1 did not influence the migration and invasion abilities of MDA-MB-231 cells. These results are consistent with the finding that migration and invasion abilities were significantly inhibited in miR-193a-3p mimic-transfected MDA-MB-231 cells but not in miR-193a-5p mimic-transfected cells.

### Target genes involved in arm expression preference of miR-193a

**Figure 5:**
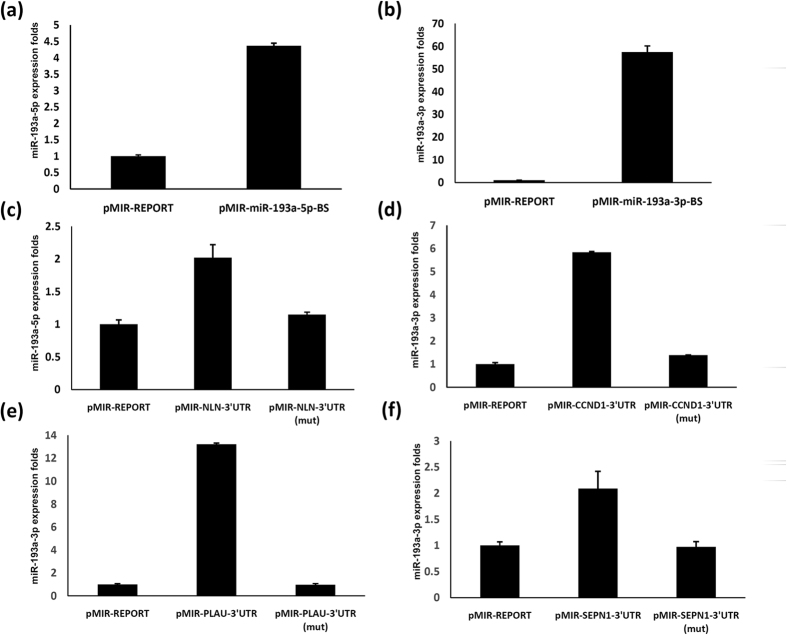
The expression levels of individual target genes could alter arm expression preference of miR-193a in breast cancer cells. (**a**) The expression levels of miR-193a-5p were increased in breast cancer cells transfected with pmiR-193a-5p (artificial target of miR-193a-5p). (**b**) The expression levels of miR-193a-3p were increased in breast cancer cells transfected with pmiR-193a-3p (artificial target of miR-193a-3p). (**c**) The expression levels of miR-193a-5p were examined after pMIR-NLN-3′UTR and pMIR-NLN-3′UTR(mut) transfection. (**d**–**f**) The expression levels of miR-193a-3p were analyzed after pMIR-CCND1-3′UTR, pMIR-CCND1-3′UTR(mut), pMIR-PLAU-3′UTR, pMIR-PLAU-3′UTR(mut), pMIR-SEPN1-3′UTR and pMIR-SEPN1-3′UTR(mut) transfection.

We investigated the reason for changes in the expression preference of miR-193a-5p and miR-193a-3p in breast cancer. Up to now, the arm selection change seems to be attributable to the thermodynamic properties of the miR–miR* duplex. However, numerous studies have reported that the miR#-5p/miR#-3p ratio varies among cell types or during carcinogenesis; therefore, the thermodynamic model alone is insufficient to explain the arm selection change^6^,^8^,^9^,^10^,^11^,^14^,^31^. Recently, the concept of target-mediated miRNA protection (TMMP) was proposed by Chatterjee *et al*.^31^. They hypothesised that the high mRNA abundance of target genes can block miRNA release, protecting it from degradation by exoribonucleases^31^,^32^; this finding suggests that miR#-5p/miR#-3p ratios are altered depending on target expression levels during the development stage or cancer progression or vary among tissue types. To test the TMMP effect *in vivo*, 5 tandem binding sites of miR-193a-5p and miR-193a-3p were cloned into the luciferase reporter vector (Supplementary Fig. S3). The reporter assay revealed that miR-193a-5p and miR-193a-3p specifically suppressed the luciferase activity of pmiR-193a-5p-BS (artificial binding site of miR-193a-5p) and pmiR-193a-3p-BS (artificial target of miR-193a-3p). Usually, miR-193a-5p expression is dominant in breast cancer (Fig. 1d). After transfection with pmiR-193a-5p-BS, the endogenous expression levels of miR-193a-5p were dramatically elevated in MDA-MB-231 cells (4–5-fold change) (Fig. 5a). The endogenous levels of miR-193a-3p (passenger arm) were also obviously increased after transfection with the pmiR-193a-3p-BS artificial target vector (60-fold change) (Fig. 5b). Moreover, we assessed the endogenous expression levels of miR-193a-5p and miR-193a-3p after transfection with the 3′UTR of NLN, CCND1, PLAU, and SNPE1. Our data revealed that endogenous levels of miR-193a-5p were slightly increased after transfection with the 3′UTR of NLN, and those of miR-193a-3p were elevated after transfection with the 3′UTR of CCND1, PLAU, and SNPE1. However, the expression levels of miR-193a-5p or -3p could not increase after transfection with the mutant 3′UTR of NLN, CCND1, PLAU, and SNPE1. (Fig. 5c–f). Considering our results together, we attribute the different miR-193a-5p/miR-193a-3p ratios to differences in the expression levels of their corresponding target genes in breast cancer.

### Arm expression preference of miRNA in breast cancer

**Figure 6:**
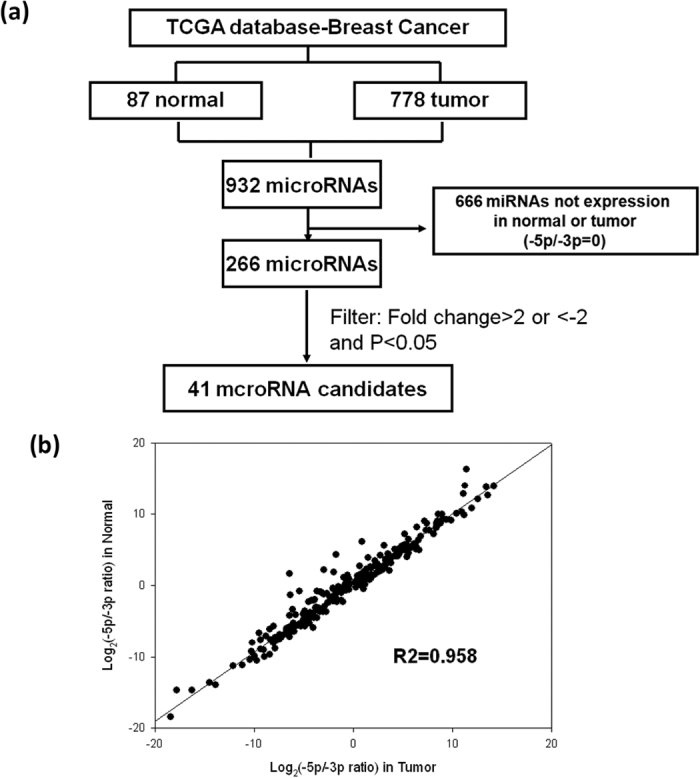
Identification of miRNA arm expression preference in breast cancer. (**a**) Workflow for the identification of miRNA arm switch candidates in breast cancer. The miRNA level-3 data of 778 breast cancer and corresponding 87 adjacent normal tissues were obtained from TCGA data portal. (**b**) A correlation was observed in the 5p/3p ratio of 266 miRNAs between breast cancer and normal tissues. The coefficient of 0.958 and *P* < 0.001 indicated that the miR-193a-5p/miR-193a-3p ratios of 266 miRNAs in breast cancer were highly correlated with those in normal tissues.

We used TCGA data to analyse the miRNA arm expression preference in breast cancer. We downloaded the small RNA-seq data of 778 breast cancer tissues and 87 adjacent normal tissues from TCGA database. Analysis of miRNA profiles revealed that 266 miRNAs possessed mature miRNA expression from both the 5p and 3p arms (>1 Transcripts Per Million) in breast cancer. We also analysed the expression levels of miR#-5p and miR#-3p arms, observing that the arm expression preference of most miRNAs was consistent between breast cancer and adjacent normal tissues (r = 0.958). Differences were observed in the arm expression preference of only a few miRNAs between breast cancer and adjacent normal tissues (>2-fold change) (Supplementary Table S1 and Fig. 6). According to our present results, we conclude that the arm expression preference of most miRNAs is determined on the basis of the hydrogen bonding selection mechanism, but this mechanism is insufficient to explain the arm expression preference of all miRNAs.

**Figure 7:**
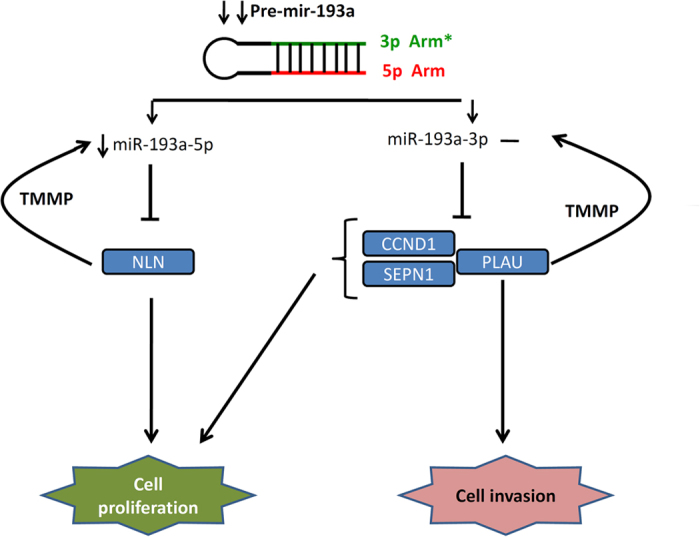
Working model for the role of dysfunctional miR-193a in the modulation of the growth and motility of breast cancer cells.

Herein, we concluded that the expression of pri-mir-193a was decreased in breast cancer cells, and the expression level of miR-193-5p/miR-193a-3p was inconsistent with pri-miR-193a expression in breast cancer. MiR-193a-5p suppressed cell growth by repressing NLN in breast cancer. Furthermore, miR-193a-3p suppressed cell growth by inhibiting CCND1, PLAU, and SEPN1 and regulated cell motility by suppressing PLAU expression. The mRNA abundance of these target genes could protect miR-193a-5p and miR-193a-3p from degradation, resulting in the difference in the arm expression preference of miR-193a in breast cancer compared with normal tissues (Fig. 7).

## Discussion

Mature miRNAs are derived from the 5p and 3p arms of the precursor during the maturation process. The expression preference of miR#-5p and miR#-3p is determined by the Ago protein on the basis of the hydrogen bonding mechanism; therefore, the arm expression preference of most miRNAs should be consistent^33^. However, a few studies have shown significant differences in the arm selection preferences of some miRNAs among tissues, developmental stages, species, and cancer types^8^,^9^,^10^,^11^,^14^. Hu *et al*. reported that more than half of pre-miRNAs showed differences in arm selection among tissue types in mice^34^. In the present study, we comprehensively analysed the arm expression preferences of miRNAs in breast cancer, observing significant differences in the arm expression preference of 41 miRNA candidates between breast cancer and normal tissues. Among them, arm switching was identified for miR-296 and miR-193a in breast cancer tissues from another independent cohort in our previous study^9^. Choo *et al*. reported that miR#-5p and miR#-3p arms are frequently coexpressed and coordinately regulated in colon cancer, implying that the arm expression preference of most miRNAs is consistent in colon cancer. However, they also reported that the 5p and 3p arms of let-7 and miR-200b are inversely regulated in colon cancer^35^. This observation is similar to our finding that only a few miRNAs exhibited changes in arm expression preferences in breast cancer (Supplementary Table S1).

In this study, we demonstrated significant differences in the miR-193a arm expression preferences in human breast cancer tissues compared with corresponding adjacent normal tissues. Moreover, we identified only a few putative target genes for miR-193a-5p but numerous putative target genes for miR-193a-3p by combining data from microarray experiments and the target prediction tool (Fig. 3). We also demonstrated that the mRNA abundance of target genes can stabilise typically degraded miRNA* sequences (Fig. 5); thus, the TMMP hypothesis may explain the arm expression preferences of miR-193a in breast cancer cells. Therefore, increasing miR-193a-3p stabilisation may have resulted in no reduction in the expression levels of miR-193a-3p (Fig. 1c). Therefore, determining the biological function of miRNA on the basis of its expression level may be not appropriate.

Because the biological function of miRNAs is dependent on the degree of complementary pairing between miRNAs and mRNAs, miRNAs exhibit a tumour suppressive or oncogenic role during cancer progression. In general, miR#-5p and miR#-3p arms have different sequences; therefore, they can modulate t different arget genes. Our data revealed that both miR-193a-5p and miR-193a-3p suppressed breast cancer cell growth, but only miR-193a-3p inhibited breast cancer cell motility (Fig. 2). According to miRbase data, miR-193a-3p expression is dominant in human tissue. This is inconsistent with our observation that the expression levels of miR-193a-5p were higher than those of miR-193a-3p in breast cancer. However, only a few studies have reported the biological function of miR-193a-5p in human cancers. Yang *et al*. indicated that miR-193a-5p inhibit tumour development by suppressing cancer cell growth and migration via the miR-193a-5p-YY1-APC axis in human endometrioid endometrial adenocarcinoma^29^. Yu *et al*. reported that both miR-193a-5p and miR-193a-3p suppressed lung cancer cell migration and invasion by coregulating the ERBB4/PIK3R3/mTOR/S6K2 signalling pathway^27^. In the present study, we showed that miR-193a-5p suppressed breast cancer cell growth but did not influence the migration ability (Fig. 2). A previous study reported that miRNA may play contrasting dual functions depending on its target gene expression in different cancer types^30^. miR-193a-5p regulates cell survival and chemosensitivity by repressing TP73 expression in squamous cell carcinoma, but it exerts no effect in bladder cancer^15^,^36^. Taken together, these inconsistent results may due to differences in cancer type.

Numerous studies have indicated that miR-193a-3p exhibits a tumour suppressive function in human cancers, including bladder cancer, hepatocellular carcinoma, non–small-cell lung cancer, pleural mesothelioma, and colon cancer^15^,^16^,^20^,^21^,^22^,^23^,^24^,^25^,^26^,^27^,^28^. Particularly in bladder cancer, Zhu’s laboratory reported the detailed mechanism underlying the promotion of multichemoresistance of bladder cancer by miR-193a-3p. They demonstrated that miR-193a-3p modulates the chemoresistance-associated signalling pathway by directly suppressing SRSF2, HIC2, HOXC9, PSEN1, LOXL4, ING5, c-kit, and PLAU^15^,^22^,^23^,^24^,^28^,^37^,^38^. Salvi *et al*. showed that miR-193a-3p was significantly downregulated in hepatocellular carcinoma, and that the ectopic expression of miR-193a-3p reduced cell proliferation and increased apoptosis by targeting uPA genes^21^. Furthermore, miR-193a-3p suppressed MCL1 by directly targeting the 3′UTR of MCL-1, suppressing cell growth and promoting apoptosis in lung cancer and malignant pleural mesothelioma^16^,^26^. In addition, Iliopoulos *et al*. reported that miR-193a-3p contributed to cellular transformation and invasive growth by directly targeting the 3′UTR of PLAU and K-Ras genes^20^. In this study, we demonstrated that miR-193a-3p suppressed breast cancer cell growth by directly inhibiting CCND1, PLAU and SEPN1 expression, and suppressed breast cancer cell migration/invasion via silencing PLAU. Herein, our data revealed that miR-193a-3p obviously suppressed breast cancer cell migration and invasion (Fig. 2). However, the PLAU knockdown only partially inhibited breast cancer cell migration and invasion (Fig. 4). This discrepancy in the results may be due to miR-193a-3p suppressing breast cancer cell motility by inhibiting several target genes. Until now, several target genes of miR-193a-3p have been identified, including MCL-1, SRSF2, PLAU, PSEN1, HOXC9, H-Ras, HIC2, LOXL4, ING5, and c-KIT^15^,^22^,^23^,^24^,^28^,^37^,^38^. Except for PLAU, previous studies have reported that PSEN1, MCL-1, and HCXC9 significantly promote cell migration and invasion^39^,^40^,^41^,^42^.

In summary, the arm expression preference phenomenon may be a means for modulating miRNA function, further complicating the mRNA regulatory network. Our findings suggest that the arm expression preference of miRNA is remarkably prevalent in breast cancer and thus provide a new insight into breast cancer progression.

## Methods

### Clinical samples

Fifty-three breast cancer and corresponding adjacent normal breast tissue samples were collected from breast cancer patients who underwent surgery at the Department of Surgery, Kaohsiung Veterans General Hospital, Kaohsiung, Taiwan. Our study protocol was independently reviewed and approved by the Institutional Review Board of Kaohsiung Veterans General Hospital (IRB approval number: VGHKS-CT12-16). The methods were carried out in accordance with the approved guidelines and all patients provided informed consent.

### Cell lines

Two human breast cancer cell lines, MDA-MB-231 and MCF7, were obtained from the American Type Culture Collection. These cells were maintained in Dulbecco’s modified Eagle’s medium (Biological Industries, IL, USA) supplemented with 10% fetal bovine serum (HyClone, USA) and penicillin-streptomycin (penicillin at 100 units/mL and streptomycin at 100 μg/mL) (Sigma-Aldrich Co. LLC., USA).

### Samples and RNA extraction

Total RNA was extracted from clinical tissues or cell lines by using TRIzol reagent (Invitrogen, Carlsbad, CA, USA) according to the instruction manual. Briefly, tissue samples were homogenised in 1 mL of TRIzol reagent and mixed with 0.2 mL of chloroform for protein extraction, and RNA was precipitated by adding 0.5 mL of isopropanol. The concentration, purity, and amount of total RNA were determined using the Nanodrop 1000 spectrophotometer (Nanodrop Technologies Inc., USA).

### Stem-loop reverse transcription and real-time polymerase chain reaction

A total of 1 μg of total RNA was reverse-transcribed in a stem-loop reverse transcription (RT) reaction by using RT primers and SuperScript III Reverse Transcriptase according to the user’s manual (Invitrogen, Carlsbad, CA, USA). The reaction was performed under the following incubation conditions: 30 min at 16 °C, followed by 50 cycles of 20 °C for 30 s, 42 °C for 30 s, and 50 °C for 1 s. The enzyme was subsequently inactivated through incubation at 85 °C for 5 min. Real-time polymerase chain reaction (PCR) was performed using a miRNA-specific forward primer and a universal reverse primer and involved incubation at 94 °C for 10 min, followed by 40 cycles of 94 °C for 15 s and 60 °C for 32 s. The gene expression level was detected using the SYBR Green I assay (Applied Biosystems, Foster City, CA, USA), and the expression levels of miRNAs were normalised to those of U6. The primer sequences used to examine miRNAs are listed in Supplementary Table S2.

### Ectopic expression of miR-193a-5p and miR-193a-3p through transfection with mimics

Breast cancer cells were seeded in a 25T flask at a density of 1 × 10^6 ^cells/mL, and these cells were transfected with 10 nM miRNA-193a-5p mimics, miR-193a-3p mimics, or the appropriate miRNA mimic control (GenDiscovery Biotechnology Inc., Taiwan) by using the Lipofectamine RNAiMAX reagent (Invitrogen, Carlsbad, CA, USA). Twenty-four hours posttransfection, the transfected cells were harvested, and the expression levels were examined using stem-loop RT-qPCR.

### Cell proliferation and colony formation assay

In the clonogenic assay, breast cancer cells were seeded in a 6-well plate at 4 × 10^3^ and transfected with 10 nM miRNA-193a-5p mimics, miRNA-193a-3p mimics, siRNA, or a control. The cells were incubated in a CO_2_ incubator at 37 °C for 2 wk until the formation of colonies with substantial sizes. The medium was removed, cells were fixed in 1 mL of 10% formaldehyde solution, and the plates were incubated at room temperature (RT) for 2 min. After removal of the fixation solution, 1 mL of crystal violet solution was added, and the plates were incubated at RT for 2 h. Subsequently, crystal violet solution was removed, and the plates were rinsed. The plates were air-dried at RT. Subsequently, 1 mL of 10% acetic acid was added to each well to dissolve the crystal violet. The absorbance of individual wells was determined at 595 nm by using Multiskan FC (Thermo Scientific, USA).

### Cell migration and invasion assays

The migration and invasion abilities of cells were assessed *in vitro* by using the Transwell assay, as previously described^43^. Briefly, cells were resuspended at a density of 4.5 × 10^5^ in 2% fetal bovine serum and then added to the upper chamber of the Transwells (Falcon, Corning Incorporated, USA) without Matrigel (BD Biosciences, MA) for the migration assay or with a Matrigel coating for the invasion assay. Chambers were incubated in a CO_2_ incubator at 37 °C for 12 or 24 h; the remaining cells in the upper chamber were removed using cotton swabs, and the cells on the undersurface of the Transwells were fixed in 10% formaldehyde solution. Cells were stained with crystal violet solution, and the numbers of breast cancer cells in 3 fields were counted under a phase-contrast microscope. All experiments were repeated 3 times.

### Microarray analysis

The cDNA probes were derived from paired RNA samples from cells transfected with miR-193a-5p mimics or a control and cells transfected with miR-193a-3p mimics or a control. The DNA probes were applied to the sample microarray chip after being labelled with the Cy3-dCTP (green) or Cy5-dCTP (red) dye. The microarray experiments and data analysis were performed using the Agilent Oligo Chip by Welgene Biotech (Taipei, Taiwan).

### Candidate target genes of miRNA and luciferase activity assay

Targets of miRNAs were predicted using the TargetScan tool and the microarray approach. The 3′UTR sequences and seed region mutant of candidate genes or artificial binding sites were cloned into the pMIR-REPROT^TM^ vector (Biotools, USA). Subsequently, breast cancer cells were cotransfected with candidate-containing pMIR-REPROT^TM^ vectors and miR-193a-5p, miR-193a-3p, or control oligonucleotides by using the Lipofectamine RNAiMAX reagent. After 24-h transfection, the luciferase activity of cell lysates was measured using the Dual-Glo Luciferase Reporter Assay System (Promega, Madison, WI, USA).

### Knockdown of gene expression by using siRNA

Breast cancer cells were transfected with either of the individual RNAi oligonucleotides directed against NLN, CCND1, PLAU, SEPN1 and random sequence siRNA oligonucleotides (Invitrogen, Carlsbad, CA, USA) were used as a negative control. Forty-eight hours after transfection, the knockdown efficiency was confirmed by western blot. The sequences of the individual siRNA oligonucleotide are listed as follows:

NLN:sense: 5′-GAUGGGAUCUUUCACCAGATT-3′

NLN:antisense: 5′-UCUGGUGAAAGAUCCCAUCTT-3′

CCND1:sense: 5′-GGAGCAUUUUGAUACCAGATT-3′

CCND1:antisense: 5′-UCUGGUAUCAAAAUGCUCCGG-3′

PLAU:sense: 5′-GCUUAACUCCAACACGCAATT-3′

PLAU: antisense: 5′-UUGCGUGUUGGAGUUAAGCCT-3′

SEPN1:sense: 5′-CCUACUUGCCGUUCACUGATT-3′

SEPN1:antisense: 5′-UCAGUGAACGGCAAGUAGGAG-3′

### Western blot

Total cell lysates were prepared using the radioimmunoprecipitation assay buffer (50 mM Tris-HCl at pH 8.0, 150 mM NaCl, 1% NP-40, 0.5% deoxycholic acid, and 0.1% sodium dodecyl sulphate). Total protein was separated through 6–10% sodium dodecyl sulphate-polyacrylamide gel electrophoresis and transferred to nitrocellulose filter membranes (Millipore, Billerica, USA). Subsequently, the membranes were blocked with a blocking buffer for 1 h at RT and incubated with the following primary antibodies: NLN (ab119802, Abcam), CCND1 (RM-9104-S, Thermo Scientific), PLAU (ab24121, Abcam) , SEPN1(sc-365824, Santa Cruz) , Actin (MAB1501, Millipore) and GAPDH (GTX627408, GeneTex) for overnight at 4 °C. Secondary labelling was performed using suitable horseradish peroxidase-conjugated secondary antibody for 1 h at RT. Finally, proteins were visualised using WesternBright^TM^ enhanced chemiluminescence (Advansta Inc., USA) and detected using the BioSpectrum^TW^ 500 Imaging System (UVP, USA).

### Expression data from The Cancer Genome Atlas

All level-3 expression data of breast cancer were downloaded from The Cancer Genome Atlas (TCGA) data portal (https://tcga-data.nci.nih.gov/tcga/dataAccessMatrix.htm). The miRNA level-3 data of 778 breast cancer tissues and 87 corresponding adjacent normal tissues were obtained from TCGA data portal. In addition, the level-3 data of the RNA transcriptome profile from 779 breast cancer tissues and 100 corresponding adjacent normal tissues were obtained from TCGA data portal.

### Statistical analysis

The expression levels of miR-193a in various cancer types from the TCGA database were analyzed using Student’s *t* tests. The data of pri-mir-193a, miR-193a-5p, or miR-193a-3p expression in paired breast tissues were analyzed using a paired *t* test. The experiments of the luciferase reporter assay, cell viability, migration, and invasion were conducted in triplicate. Histograms present the means values, and the error bars indicate the SD. These data were analyzed using Student’s *t* tests. The correlation of the miR-193a-5p/-3p ratio between the breast cancer and normal tissues was determined through Pearson’s coefficient analysis, with *r* and P values as indicated. The difference was considered significant where *p* < 0.05.

## Additional Information

**How to cite this article**: Tsai, K.-W. *et al*. Arm Selection Preference of MicroRNA-193a Varies in Breast Cancer. *Sci. Rep.* **6**, 28176; doi: 10.1038/srep28176 (2016).

## Supplementary Material

Supplementary Information
