# Supplementary material for: Arm Selection Preference of MicroRNA-193a Varies in Breast Cancer
Source: Sci Rep. 2016 Jun 16;6:28176. doi: 10.1038/srep28176 (PMC4910092; doi:10.1038/srep28176)
Supplement: Supplementary Information [file srep28176-s1.doc]

**Arm Selection Preference of MicroRNA-193a Varies in Breast Cancer**

**Kuo-Wang Tsai^1,2#^, Chung-Man Leung^3#^ ,** [**Yi-Hao Lao**](http://www.sciencedirect.com/science/article/pii/S0735675714004549)**^4#^ Ting-Wen Chen ^5, 6^, Wen-Ching Chan^7^, Shou-Yu Yu^1^, Ya-Ting Tu^1^ ,Hing-Chung Lam^8^, Sung-Chou Li^7^, Luo-Ping Ger^1,9^, Wen-Shan Liu^3,10*^ and Hong-Tai Chang ^8,11*^**

^1^ Department of Medical Education and Research, Kaohsiung Veterans General Hospital, Kaohsiung, Taiwan

^2^ Department of Chemical Biology, National Pingtung University of Education, Pingtung, Taiwan

^3^ Department of Radiation Oncology, Kaohsiung Veterans General Hospital, Kaohsiung, Taiwan

^4^ Department of Family Medicine, Zuoying Branch of Kaohsiung Armed Forces General Hospital, Kaohsiung, Taiwan

^5^Molecular Medicine Research Center, Chang Gung University, Taoyuan, Taiwan

^6^Bioinformatics Center, Chang Gung University, Taoyuan, Taiwan

^7^Genomics & Proteomics Core Laboratory, Department of medical research, Kaohsiung Chang Gung

^8^Center For Geriatrics and Gerontology, Kaohsiung Veterans General Hospital, Kaohsiung, Taiwan

Memorial Hospital and Chang Gung University College of Medicine, Kaohsiung, Taiwan

^9^Institute of Biomedical Sciences, National Sun Yat-Sen University, Kaohsiung, Taiwan

^10^ Department of Radiation Oncology, Tri-Service General Hospital

^11^ Department of Surgery, Kaohsiung Veterans General Hospital, Kaohsiung, Taiwan

**Supplementary Figure 1. Potential targets were measured in MDA-MB-231 or MCF-7 cells transiently transfected with miR-193a-5p mimics, miR-193a-3p mimics, or control oligonucleotides**

(**a**) NLN expression was examined after transfection of MAD-MB-231 (left panels) and MCF-7 (right panels) cells with miR-193a-5p mimics. (**b**) The expression of 10 putative targets of miR-193a-3p was examined using real-time PCR after transfection of MAD-MB-231 (upper panel) and MCF-7 (below panel) cells with miR-193a-3p mimics. (**c**) The levels of the DCAF7 protein were examined using western blotting after transfection of MDA-MB-231 and MCF-7 cells with miR-193a-3p mimics.

**Supplementary Figure 2. The PLAU is a direct target of miR-193a-3p in breast cancer cells.** Schema of the luciferase constructs. The miR-193a target sequence in the 3'UTR region of PLAU is shown in the upper panel and the mutant of its 3'-UTR is shown in red. Relative luciferase activity of the reporter with the 3'UTR and 3'UTR(mut) of PLAU genes was determined after cotransfection of breast cancer cells with miR-193a-3p mimics. Firefly luciferase activity served as a normalisation control.

**Supplementary Figure 3**

(**a**) The sequences of artificial targets of miR-193a-5p and miR-193a-3p are shown. (**b**) and (c) Relative luciferase activity of reporters with artificial target genes was determined after cotransfection of breast cancer cells with miR-193a-5p and miR-193a-3p mimics. Firefly luciferase activity served as a normalisation control.

**Supplementary Figure 1**

**
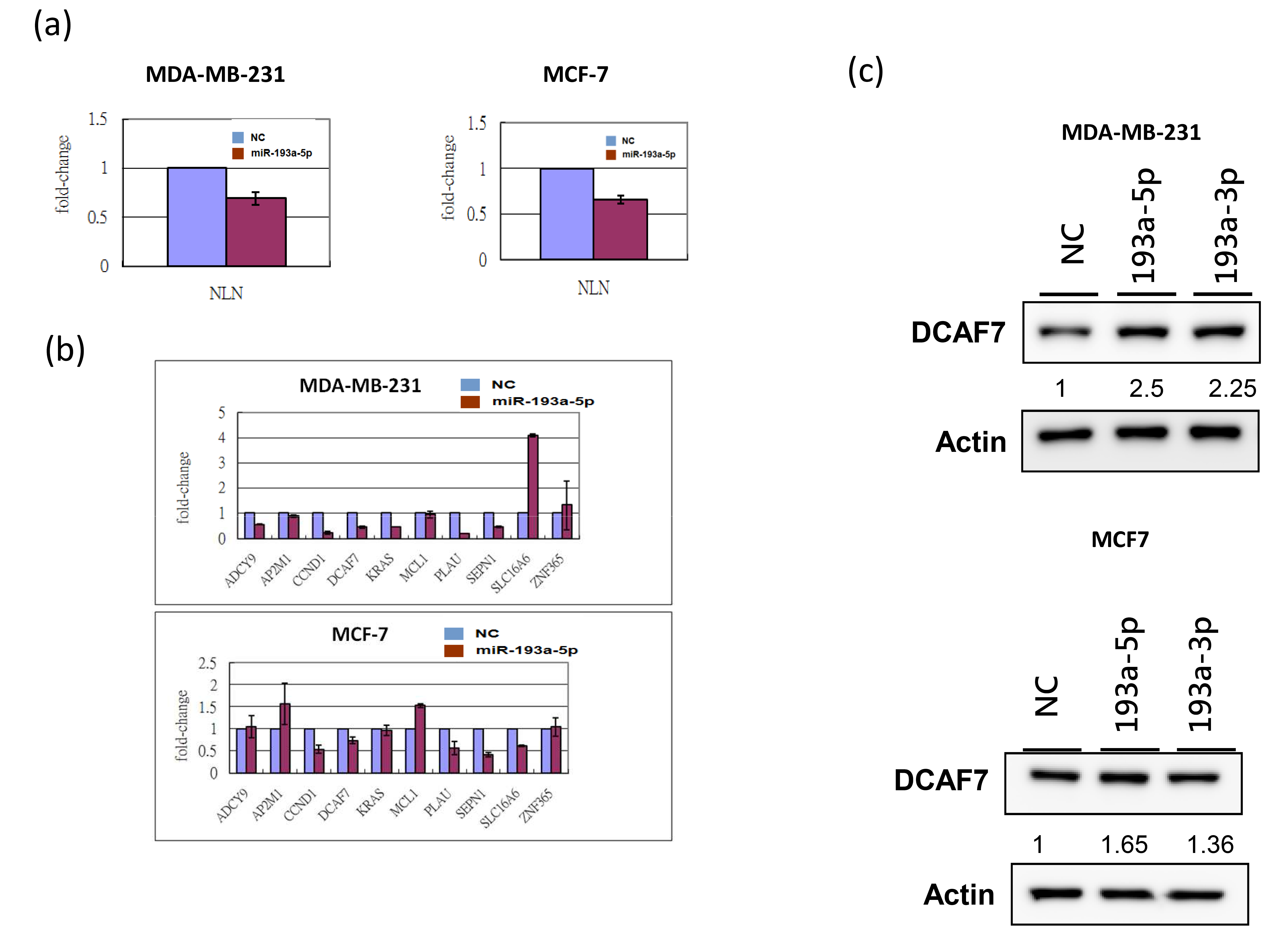
**

**Supplementary Figure 2**

**Supplementary Figure 3**

**
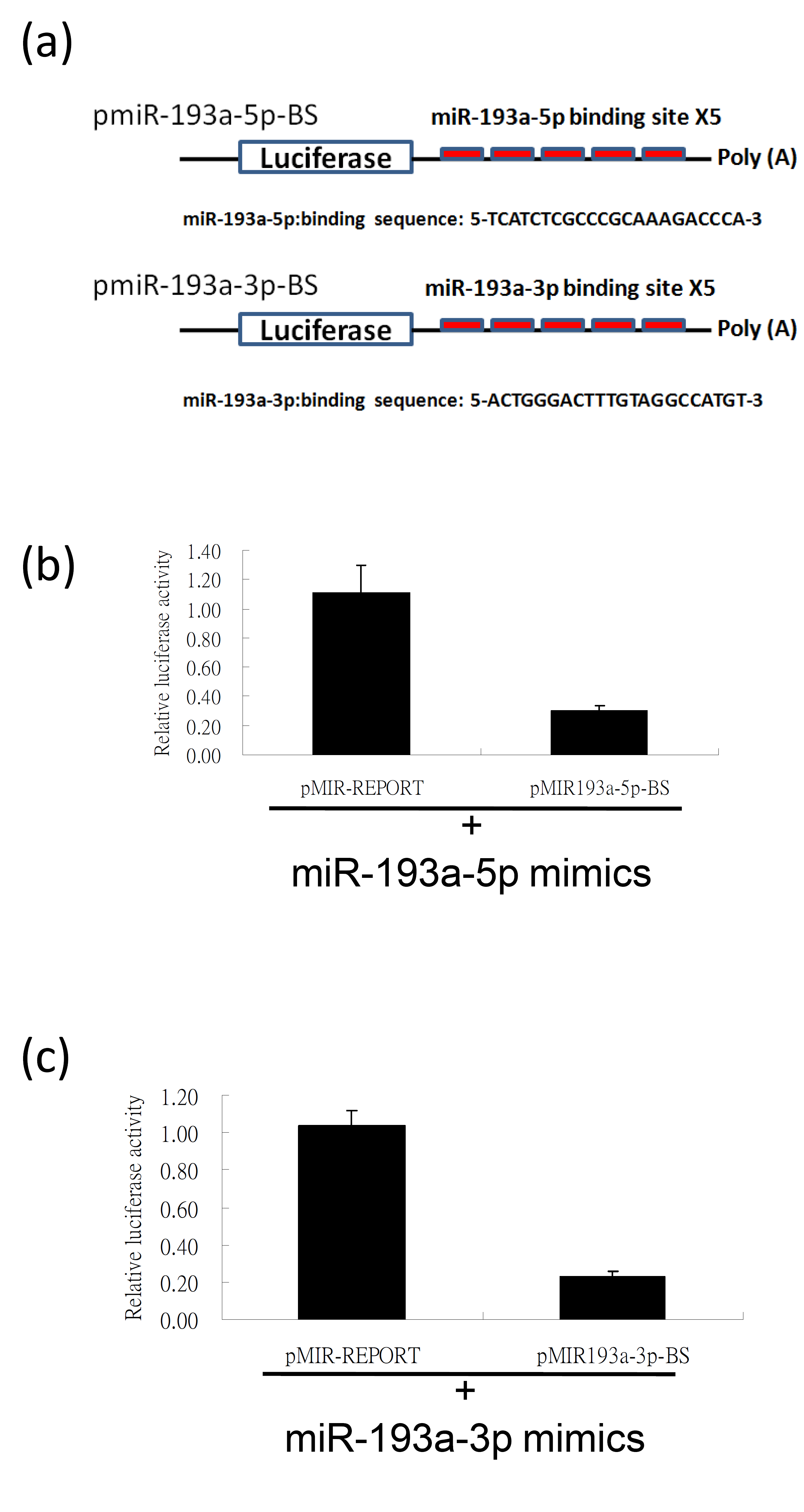
**

| **Supplementary Table S1. The miRNA candidates shows arm selection preferences change in breast cancer compared to normal tissues** | | | | |
| --- | --- | --- | --- | --- |
| **Gene_symbol** | **normal (5p/ 3p)** | **Tumor (5p/3p)** | **ratio(T/N)** | **pvalue** |
| **has-miR-885** | 0.01149 | 3.225 | 280.6788512 | 0.00008276 |
| **has-miR-105** | 0.3103 | 20.03 | 64.55043506 | 0.0003074 |
| **has-miR-96** | 1.897 | 71.25 | 37.55930416 | 2.2E-16 |
| **has-miR-767** | 0.1264 | 4.652 | 36.80379747 | 0.00004421 |
| **has-miR-449b** | 0.01245 | 0.3843 | 30.86746988 | 2.2E-16 |
| **has-miR-182** | 2796 | 77740 | 27.80400572 | 2.2E-16 |
| **has-miR-19b** | 0.2644 | 3.759 | 14.21709531 | 0.0001056 |
| **has-miR-2114** | 0.08046 | 0.5813 | 7.224707929 | 3.991E-15 |
| **has-miR-30c** | 0.01461 | 0.09405 | 6.437371663 | 6.695E-09 |
| **has-miR-196a** | 8.923 | 50.31 | 5.638238261 | 2.2E-16 |
| **has-miR-135b** | 2.784 | 15.31 | 5.499281609 | 8.721E-11 |
| **has-miR-545** | 0.1034 | 0.5484 | 5.303675048 | 0.0000041 |
| **has-miR-138** | 0.01149 | 0.05527 | 4.8102698 | 0.02943 |
| **has-miR-624** | 0.1092 | 0.5105 | 4.674908425 | 0.001899 |
| **has-miR-26a** | 0.04563 | 0.2091 | 4.582511506 | 3.445E-09 |
| **has-miR-431** | 0.05483 | 0.2392 | 4.362575233 | 2.2E-16 |
| **has-miR-519a** | 0.5172 | 2.205 | 4.263341067 | 0.0003513 |
| **has-miR-33b** | 1.583 | 6.679 | 4.219204043 | 2.2E-16 |
| **has-miR-149** | 37.66 | 143.5 | 3.810408922 | 2.978E-15 |
| **has-miR-877** | 0.6916 | 2.632 | 3.805668016 | 4.32E-15 |
| **has-miR-7** | 0.01757 | 0.05953 | 3.388161639 | 1.91E-10 |
| **has-miR-15a** | 87.99 | 286.6 | 3.257188317 | 2.839E-10 |
| **has-miR-3678** | 0.2414 | 0.7842 | 3.248550124 | 0.00007935 |
| **has-miR-671** | 0.6908 | 2.122 | 3.071800811 | 2.2E-16 |
| **has-miR-34b** | 0.2437 | 0.7207 | 2.957324579 | 2.2E-16 |
| **has-miR-556** | 0.1621 | 0.4611 | 2.844540407 | 0.00001724 |
| **has-miR-3613** | 6.687 | 18.23 | 2.726185135 | 2.2E-16 |
| **has-miR-188** | 0.7447 | 1.928 | 2.588961998 | 1.422E-12 |
| **has-miR-106b** | 0.3533 | 0.8689 | 2.459382961 | 2.2E-16 |
| **has-miR-2277** | 0.6299 | 1.528 | 2.42578187 | 3.557E-08 |
| **has-miR-33a** | 4.516 | 10.75 | 2.380425155 | 2.2E-16 |
| **has-miR-3605** | 0.03055 | 0.06222 | 2.036661211 | 0.0415 |
| **has-miR-138** | 0.3266 | 0.6536 | 2.00122474 | 0.004773 |
| **has-miR-144** | 49.17 | 22.83 | 0.464307505 | 0.000007568 |
| **has-miR-202** | 2.351 | 1.081 | 0.459804339 | 0.01515 |
| **has-miR-497** | 81.72 | 34.86 | 0.426578561 | 0.00002214 |
| **has-miR-488** | 0.4939 | 0.2008 | 0.406560032 | 0.007801 |
| **has-miR-369** | 2.083 | 0.7376 | 0.354104657 | 1.075E-10 |
| **has-miR-296** | 12.78 | 4.392 | 0.343661972 | 5.027E-07 |
| **has-miR-193a** | 99.07 | 31.92 | 0.322196427 | 0.0000022 |
| **has-miR-27a** | 0.06198 | 0.01638 | 0.2642788 | 1.414E-09 |

**Supplementary Table S2: primer sequence list**

| **primer name** | **sequence** |
| --- | --- |
| **miR-193a-3p-RT** | CTCAACTGGTGTCGTGGAGTCGGCAATTCAGTTGAGACTGGGAC |
| **miR-193a-5p-RT** | CTCAACTGGTGTCGTGGAGTCGGCAATTCAGTTGAGTCATCTCG |
| **miR-1963a-3p-GSF** | CGGCGGAACTGGCCTACAAAGT |
| **miR-193a-5p-GSF** | CGGCGGTGGGTCTTTGCGGGCG |
| **U6-F** | CTC GCT TCGGCAGCA CA |
| **U6-R** | AACGCTTC CGAATTTGCGT |
| **Universal-R** | CTGGTGTCGTGGAGTCGGCAATTC |
| **NLN-F** | CAAACTACTCGGTTATAGCAC |
| **NLN-R** | CATCTAGAAAGGCTGTTACG |
| **ADCY9-F** | CCCAGACAGTTCTGTATTAAC |
| **ADCY9-R** | AGGAGAAAGAAGACGAGAAC |
| **AP2M1-F** | GAGGAGGGGCCTAGAGTCAT |
| **AP2M1-R** | ACTGTGTTAGGACAAGCCGAG |
| **CCND1-F** | GCCTCTAAGATGAAGGAGAC |
| **CCND1-R** | CCATTTGCAGCAGCTC |
| **DCAF7-F** | AGGAGATCTACAAGTATGAAGC |
| **DCAF7-R** | GAACCTTGTTGTTGTACTCC |
| **KRAS-F** | GCCTGCTGAAAATGACTG |
| **KRAS-R** | TCCTGTAGGAATCCTCTATTG |
| **PLAU-F** | GAAAACCTCATCCTACACAAG |
| **PLAU-R** | ATTCTCTTTTCCAAAGCCAG |
| **SEPN1-F** | TCTACTACACTGTGATGTTCC |
| **SEPN1-R** | TCTTTGGAGAGGATGATGTG |
| **SLC16A6-F** | AATGAAACAGCTACCGCCCA |
| **SLC16A6-R** | TCATCGATCCTCATGCTATTGCT |
| **ZNF365-F** | AGAAAAAGCAGGAAGTTCAG |
| **ZNF365-R** | CTTCTTCTTTCCTAAGAAGCTC |
